# Fumes of Nitrate of Potass in Spasmodic Asthma

**Published:** 1845-07

**Authors:** James Bower Harrison


*Fumes of Nitrate of Potass in Spasmodic Asthma.* By James Bower Harrison, M. R. C. S., &c. &c.—When attention was first directed to the nature of the atmosphere, and the influence of the different gases on the respiratory organs, much advantage was expected from the application of the discoveries which were made, but in this, as in many other departments of knowledge, the immediate results were not so brilliant as was anticipated. It is probable, however, that medicated pneumatic mixtures will eventually make a considerable accession to our remedial measures.

A friend of mine, who is the subject of spasmodic asthma, having accidentally seen, in a Staffordshire paper, a paragraph recommending the inhalation of the fumes of nitrate of potass as a remedy for that complaint, was induced to make trial of it. He had previously employed various remedies, but with only very partial and temporary relief. The experiment was attended with the most complete succes. He made use of a piece of blotting paper, about the size of his hand, dipped in a saturated solution of the nitrate of potass, and afterwards dried. This was placed on a common plate, and being ignited, the fumes were speedily sensible in the room. He described its operation "as clearing the passages and gradually opening the air tubes." He said that the respiratory murmur became louder and less stridulous, under its continued influence. The effect was always produced in about a quarter of an hour, and though he had used the same remedy nearly twenty times, he had never been disappointed in the result. On one occasion, when it was adopted in a larger room than customary, he did not (as might be expected) experience so beneficial an effect. As I have had many opportunities of ascertaining the urgency and frequency of the asthmatic paroxysms in this gentleman's case, and can place every reliance on the veracity of his statement, I think it may be of advantage to put his case on record, more especially as I imagine the remedy to be as yet but little known, or fairly appreciated. It may be intersting to notice, that another member of the same family is the subject of that curious complaint called the hay asthma, which was originally described by Dr. Bostock.—*London Lancit.*

